# Genes from Chagas Susceptibility Loci That Are Differentially Expressed in *T. cruzi*-Resistant Mice Are Candidates Accounting for Impaired Immunity

**DOI:** 10.1371/journal.pone.0000057

**Published:** 2006-12-20

**Authors:** Sebastian E. B. Graefe, Thomas Streichert, Birgit S. Budde, Peter Nürnberg, Christiane Steeg, Bertram Müller-Myhsok, Bernhard Fleischer

**Affiliations:** 1 Institute for Immunology, University Hospital Eppendorf Hamburg, Germany; 2 Department for Medical Microbiology and Immunology, Bernhard-Nocht-Institute for Tropical Medicine Hamburg, Germany; 3 Institute for Clinical Chemistry, University Hospital Hamburg, Germany; 4 Cologne Center of Genomics and Institute for Genetics, University of Cologne Köln, Germany; 5 Max-Planck-Institute for Experimental Psychiatry München, Germany; New York University School of Medicine, United States of America

## Abstract

Variation between inbred mice of susceptibility to experimental *Trypanosoma cruzi* infection has frequently been described, but the immunogenetic background is poorly understood. The outcross of the susceptible parental mouse strains C57BL/6 (B6) and DBA/2 (D2), B6D2F1 (F1) mice, is highly resistant to this parasite. In the present study we show by quantitative PCR that the increase of tissue parasitism during the early phase of infection is comparable up to day 11 between susceptible B6 and resistant F1 mice. A reduction of splenic parasite burdens occurs thereafter in both strains but is comparatively retarded in susceptible mice. Splenic microarchitecture is progressively disrupted with loss of follicles and B lymphocytes in B6 mice, but not in F1 mice. By genotyping of additional backcross offspring we corroborate our earlier findings that susceptibility maps to three loci on Chromosomes 5, 13 and 17. Analysis of gene expression of spleen cells from infected B6 and F1 mice with microarrays identifies about 0.3% of transcripts that are differentially expressed. Assuming that differential susceptibility is mediated by altered gene expression, we propose that the following differentially expressed transcripts from these loci are strong candidates for the observed phenotypic variation: *H2-Eα, H2-D1, Ng23, Msh5* and *Tubb5* from Chromosome 17; and *Cxcl11, Bmp2k* and *Spp1* from Chromosome 5. Our results indicate that innate mechanisms are not of primary relevance to resistance of F1 mice to *T. cruzi* infection, and that differential susceptibility to experimental infection with this protozoan pathogen is not paralleled by extensive variation of the transcriptome.

## Introduction

Chagas' disease severely affects a considerable number of persons on the American continent, but in the majority of infected, it takes an ‘indeterminate’ course over a long period of time [1]. Genetic factors determining the course and outcome of the infection are thought to be of major influence on the severity of the disease [2]–[6], but the precise background has not been elucidated. Variablity of parasite strains contributes to the complex host-pathogen interaction [3]. As in human disease, the experimental infection has an early parasitaemic phase, which is followed by chronic infection that may or may not lead to the symptoms characteristic of the disease. Some controversy has prevailed over the question whether the severity of the acute phase of the infection and the degree of parasitaemia and/or tissue parasitism correlated with the severity of the chronic complications of Chagas' disease. Recently, it has increasingly been appreciated that the persistence of parasites, rather than the occurence of autoreactive antibodies or cells, determines the degree of tissue destruction [7]–[11]. It was shown that an early phase with high parasitic loads resulted in a late phase with more prominent repercussions on the integrity of affected tissue, with more intense inflammatory infiltrates, more tissue destruction and greater loss of physiological function [12], [13].

The course of experimental *T. cruzi* infection in inbred strains of mice varies considerably depending on the mouse strain, the route of infection, the parasite strain, and the clone of a given parasite strain [14]–[16]. Other than with Leishmania, no consistent picture has evolved that would relate a certain type of immunologic reactivity with protection from severe disease. It has been noted that certain H2 haplotypes confer a degree of resistance [17]–[19]. The requirement for pro-inflammatory cytokines such as IL-12, IFN-γ, and TNF-α, as well as for MHC-class I and II molecules, CD4+ and CD8+ T lymphocytes and the synthesis of antigen-specific antibodies, for protective immunity has repeatedly been demonstrated [20]–[25]. Generally, a higher degree of expression of anti-inflammatory cytokines such as IL-4, IL-10 and TGF-β was correlated with increased severity of infection, but some conflicting results have been published (e.g., [26], [27]).

In contrast to the parental strains C57BL/6 (B6) and DBA/2 (D2), B6D2F1 (F1) hybrid mice display a considerable degree of resistance to experimental infection in terms of parasitaemia levels and rates of mortality, but precise mechanisms that explained the unusual phenotype of this strain have not been identified. By comparison with susceptible B6 mice, resistance in F1 mice was related to decreased expression of IL-10 and TGF-β in the early phase [21], [28]. However, the isolated analysis of cytokine responses, and the correlation of cytokine expression or regulatory molecules with outcome, bear the danger of focussing on secondary effects or on counter-regulative reactivity, rather than identifying the initial cause for differential outcomes. In the present work, we therefore investigated at which stage of experimental infection tissue parasite loads dissociated between susceptible B6 and resistant F1 mice in order to identify the time point at which the immune responses diverge. We then analysed genomewide expression differences at this time point in the spleen, identified transcriptional correlates for differential outcomes and matched the genomic localisation of these genes with mapped susceptibility loci.

## Results

### Experimental *T. cruzi* infection in susceptible B6 and in resistant F1 mice

**Figure 1:**
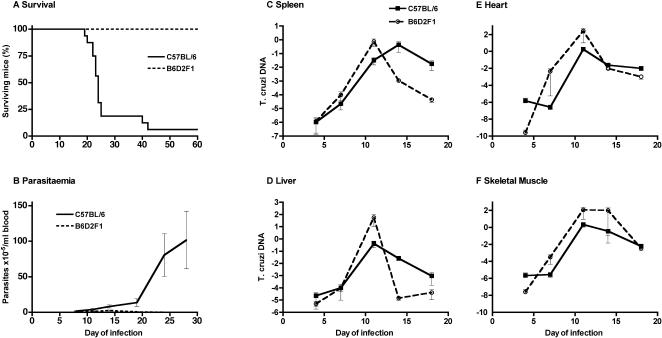
Experimental ***T. cruzi*** **infection in susceptible B6 and in resistant F1 mice.** Mice were infected with 10^4^ *T. cruzi* trypomastigotes into the peritoneum. Survival was monitored up to day 60. Parasitaemia was determined in a Neubauer chamber. Tissue parasite loads in spleen, liver, heart and skeletal muscle were determined by real time PCR and are expressed as arbitrary log units of *T. cruzi* DNA relative to quantity of murine β-actin DNA.

Infection of B6 mice with 10^4^ trypomastigotes of the *T. cruzi* tulahuen strain caused an acute illness that was usually lethal. Mice succumbed between days 20 and 30 after infection (fig. 1 A). Shortly before death, parasitaemia increased to well over 10^6^/ml (fig. 1 B). In F1 mice, there was a transient para–sitaemic phase around day 13, but parasitaemia did not exceed 10^6^/ml, and all mice invariably survived experimental infection under these conditions (fig 1 A, B). Tissue parasite loads were quantified by real time PCR in various organs during the early phase of the infection (until day 17, fig. 1 C–F). Interestingly, the degree of tissue parasitism was not significantly different in the first 11 days of infection between mouse strains. Resistant F1 mice actually displayed slightly higher parasite loads in all tested tissues up to this stage. After day 11, tissue parasite load declined in resistant mice, while it further increased in spleens of susceptible B6 mice. Thereafter, parasitism decreased in susceptible mice, too, in all tested organs. The rate of parasite clearance in the spleen was lower than in F1 mice. These results indicate that innate resistance is not compromised, if not more effective, in B6 mice. They also indicate that acquired immune responses develop in B6 mice with a delay and appear to be less efficient in containing tissue parasitism, especially in the spleen and liver.

### Splenic architecture was severely disrupted, and cellular composition was altered, in susceptible mice infected with *T. cruzi*

After day 10 of infection, there was a striking difference of parasite loads in the spleen between the mouse strains. Splenomegaly was present in all infected mice to the same extent (total spleen cell numbers increased to about 1–2×10^8^ in either strain) and pointed to a strong involvement of the spleen in the immune response to *T. cruzi*. We thus assessed by histologic analysis whether splenic architecture was modified, and by flow cytometry whether there were quantitative differences in splenic cellular composition between B6 and F1 mice.

**Figure 2:**
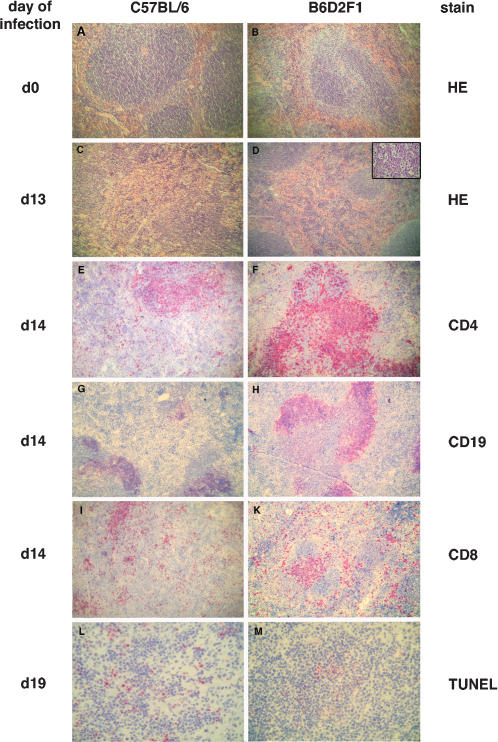
Splenic architecture and cellular composition in susceptible B6 and in resistant F1 mice. Spleens from B6 mice (left panel) and from F1 mice (right panel) were either fixed in paraformaldehyde, embedded in paraffin, and subsequently stained with hematoxylin and eosin (A–D), or they were shock frozen in liquid nitrogen and analysed by immunohistochemistry (C–M). Sections are from naive mice (A, B) or from *T. cruzi*- infected mice (C–M) at the indicated day of infection. Cryosections were stained for CD4 (E, F), CD19 (G, H), CD8 (I, K), or by the TUNEL reaction (L, M). Micrographs A–K at original magnification 100×, micrographs L, M and insert in D at 400×; inset in D shows centre of a secondary follice.

Histologically, the appearance of red and white pulp, the numbers and size of primary follicles, and the distribution of periarteriolar lymphatic sheaths (PALS) was not obviously different in naive mice, and the cellular localisation of cells expressing CD4, CD8, CD19 and CD11b was indistinguishable (fig. 2 A, B and data not shown). By day 13 of an experimental infection with *T. cruzi*, splenic microarchitecture was observed to be disrupted in B6 mice but not in F1 mice (fig. 2 C, D). The disruption was even more severe on day 23 (not shown). In F1 mice, abundant cells with intracellular apoptotic bodies, resembling tingible body macrophages, were seen in secondary follicles (fig. 2 D, inset), but these were not observed in B6 mice. Periarteriolar sheaths (PALS) were reduced by numbers and smaller in size in B6 mice, but CD4+ cells were found throughout the spleen (fig. 2 E). In F1 mice, some prominent PALS consisting of CD4+ T cells were detectable (fig. 2 F). Follicles with CD19+ B lymphocytes were also reduced in size and numbers in the spleen of infected B6 mice (fig. 2 G). Contrarily, in resistant F1 mice, prominent B cell follicles were present adjacent to PALS (fig. 2 H). CD8 T cells did generally not co-localise to PALS in susceptible mice, but were located in the remains of the red and white pulp (fig. 2 I). In F1 mice, CD8+ cells formed small clusters in the red pulp and co-localised with PALS (fig. 2 K). In summary, the main difference in splenic microarchitecture between mouse strains was the obvious loss of compartmentalised organisation of lyphocytes in susceptible but not in resistant mice.

The proliferation of *T. cruzi* within macrophages has been shown to be enhanced in the presence of apoptotic T cells due to a downregulatory effect of apoptotic bodies on trypanocidal effector functions of infected macrophages [29]. We therefore determined the quantitiy and distribution of apoptotic cells in the spleens of infected mice. In spleens from B6 mice, abundant apoptotic cells (identified by the TUNEL reaction) were found throughout the spleen, and they were frequently located sub-capsularly and in the remaining T cell areas, (fig. 2 L). By contrast, apoptotic cell numbers were lower, and they were mainly found in the centre of follicles, in the spleen of F1 mice (fig. 2 M).

**Figure 3:**
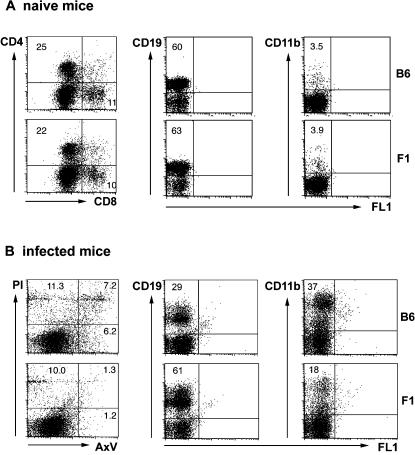
Cellular composition of the spleen from naive and *T. cruzi*- infected mice. Single spleen cell suspensions from naive mice (A) and from mice infected on day 24 with *T. cruzi* (B) were analysed by flow cytometry. Cells were stained for both CD4 and CD8, for CD19, for CD11b, or with propidium iodide (PI) and Annexin V (AxV). Numbers indicate percentage of cells expressing the respective marker. B6, C57BL/6 mice; F1, B6D2F1 mice.

The splenic cellular composition in infected mice was analysed cytometrically with explanted spleen cells to verify the histologic alterations. In naive mice, relative numbers of splenic CD4+ T cells, CD8+ T cells, CD19+ B cells and CD11b+ macrophages did not differ between mice (fig. 3 A). However, cell fractions were notably different later during infection. On day 24 of infection, prior to the detrimental outcome in B6 mice, CD19+ B lymphocytes were decreased at around 30% of cells (fig 3 B) in susceptible B6 mice. Interestingly, in infected F1 mice, the fraction of B cells was similar to that of control mice (about 60%, fig. 3 B). The fraction of CD4+ lymphocytes was 10–15% in both strains (not shown). Splenic CD8+ T cells increased to around 20–25% of cells in B6 mice, and to about 15% in F1 mice (not shown). Strikingly, in B6 mice, relative numbers of CD11b+ macrophages drastically increased more than tenfold, from about 3% in naive mice to 30–50% in infected mice, but in F1 mice, the increase was less pronounced to about 10–20% of spleen cells (fig. 3 B). In summary, a strong bias towards macrophages, and a slight bias towards CD8+ T cells, at the expense of B lymphocytes, was found in the spleen of infected B6 mice, but not in resistant F1 mice.

In naive mice, numbers of apoptotic cells were low at around 1% (determined cytometrically by staining with Annexin V) and did not differ between the two strains (not shown). In infected mice, however, there was a strong increase of Annexin V (AxV) positive cells in B6 mice (fig 3 B). The relative number of cells expressing AxV was 10–20%, with half of these also staining with propidium iodide (PI), indicating that these cells had lost integrity of the cell membrane. In infected F1 mice, AxV positive cells increased to about 2–5%. The proportion of cells stained by PI only was about 10% in either strain, indicating that preparation of samples had equal effects on staining of cells in causing some degree of cell death. In summary, susceptible mice displayed a considerably higher rate of apoptosis in the spleen, possibly accounting for the reduced numbers of B cells.

### Significant linkage of susceptibility to loci on Chromosomes 5 and 17, and suggestive linkage on Chromosome 13

**Figure 4:**
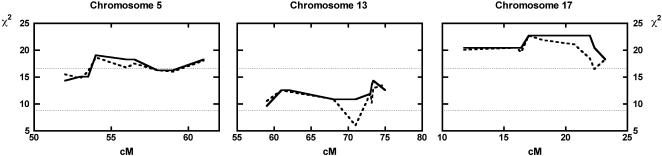
Murine *T. cruzi* susceptibility loci map to Chromosomes 5, 13, and 17. Nine microsatellite markers from the three candidate genomic regions previously identified as suceptibility loci [30] were typed in an additional 22 *T. cruzi*- susceptible B6xF1 backcross mice (see table 3 for respective markers). Single-point (dashed line) and multi-point (solid line) calculations of χ^2^ are shown for all 68 susceptible backcross mice including the 46 from the original study. Dotted lines at χ^2^ = 16.56 and χ^2^ = 8.74 indicate thresholds for suggestive and significant linkage, respectively.

We previously mapped genomic loci as being either suggestively (Chromosome 5) or significantly (Chromosome 17) linked to susceptibility to lethal experimental *T. cruzi* infection, respectively [30]. The score of a further genomic region on Chromosome 13 did not attain the threshold level for genome-wide linkage but appeared to be involved, too. In an extension study, designed to confirm linkage of a locus previously reported as suggestively linked [31], we phenotyped further male B6xF1 backcross mice, and another 22 mice died during the early phase (up to day 50) of infection. The candidate regions were genotyped with microsatellite markers in these mice, and the resulting data replicated that susceptibility mapped to the reported locus on Chromosome 17, and further substantiated the linkage evidence for the loci on Chromosomes 5 and 13 by raising it to the level of significant and suggestive linkage, respectively (fig. 4).

Even though the genes conferring susceptibility are thus mapped, the respective genetic regions are large and the number of candidate genes is substantial, so that safe predictions about the genes themselves are not straightforward. However, it is generally accepted that quantitative differences of transcription determine the outcome of an infection with a complex immunogenetic background. The kinetics of tissue parasite loads during early experimental *T. cruzi* infection in the two differentially susceptible mouse strains indicate that after about day 10, the implementation of effector immune responses was impaired but not abrogated in susceptible B6 mice. The described differences in parasite proliferation, splenic architecture and splenic cellular composition progressively developed after this time point. Since the spleen appeared to be fundamentally involved in the differential immunity to the parasite, we therefore analysed genome wide expression in explanted spleen cells from either mouse strain in order to identify genes that were differentially transcribed and that mapped to the susceptibility loci. We chose to analyse expression on day 11 as the starting point from which the infection took a divergent course. At this stage, the cellular composition of the spleen was marginally different between mouse strains in that there were more CD8+ cells (7% vs. 4%) in B6 mice, but fewer CD19+ cells, (10% vs. 18%) in B6 mice, while the proportion of CD4+ and of CD11b+ cells was comparable (12% and 17–20%, respectively).

### Differential gene expression in the spleens of susceptible B6 and from resistant F1 mice on day 11 of *T. cruzi* infection

We used mouse expression arrays containing 22690 transcripts and obtained three biologically independent specimen pairs. Data were normalised using target value scaling (target value: 100). Expression was considered significantly different when it showed a concordant regulation in at least 6 of 9 crosswise comparisons and a signal log ratio of 0.8 (fold change of 1.6) or more. Applying these criteria, we found that 28 transcripts were upregulated and 42 transcripts were downregulated in B6 mice, compared to F1 mice (tables 1 and 2). A second, biologically independent experiment was performed in which we analysed the transcription profile of pooled spleen cells from three infected animals of each strain to confirm transcriptional differences. Applying the same statistical algorithms and criteria, we could verify the differential expression of 14 transcripts with increased expression and of 35 transcripts with decreased expression (highlighted by bold gene titles, tables 1 and 2).

**Table 1:**
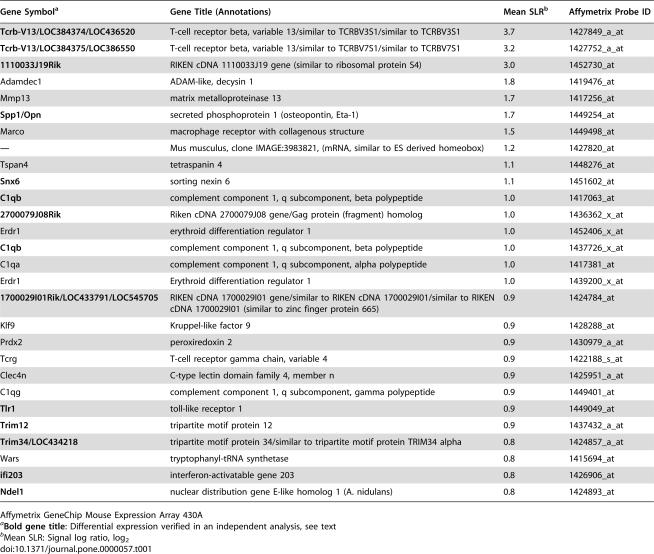
Transcripts with increased expression in spleens of *T. cruzi*- infected susceptible B6 mice with respect to resistant F1 mice.

Gene Symbol^a^	Gene Title (Annotations)	Mean SLR^b^	Affymetrix Probe ID
**Tcrb-V13/LOC384374/LOC436520**	T-cell receptor beta, variable 13/similar to TCRBV3S1/similar to TCRBV3S1	3.7	1427849_a_at
**Tcrb-V13/LOC384375/LOC386550**	T-cell receptor beta, variable 13/similar to TCRBV7S1/similar to TCRBV7S1	3.2	1427752_a_at
**1110033J19Rik**	RIKEN cDNA 1110033J19 gene (similar to ribosomal protein S4)	3.0	1452730_at
Adamdec1	ADAM-like, decysin 1	1.8	1419476_at
Mmp13	matrix metalloproteinase 13	1.7	1417256_at
**Spp1/Opn**	secreted phosphoprotein 1 (osteopontin, Eta-1)	1.7	1449254_at
Marco	macrophage receptor with collagenous structure	1.5	1449498_at
**—**	Mus musculus, clone IMAGE:3983821, (mRNA, similar to ES derived homeobox)	1.2	1427820_at
Tspan4	tetraspanin 4	1.1	1448276_at
**Snx6**	sorting nexin 6	1.1	1451602_at
**C1qb**	complement component 1, q subcomponent, beta polypeptide	1.0	1417063_at
**2700079J08Rik**	Riken cDNA 2700079J08 gene/Gag protein (fragment) homolog	1.0	1436362_x_at
Erdr1	erythroid differentiation regulator 1	1.0	1452406_x_at
**C1qb**	complement component 1, q subcomponent, beta polypeptide	1.0	1437726_x_at
C1qa	complement component 1, q subcomponent, alpha polypeptide	1.0	1417381_at
Erdr1	Erythroid differentiation regulator 1	1.0	1439200_x_at
**1700029I01Rik/LOC433791/LOC545705**	RIKEN cDNA 1700029I01 gene/similar to RIKEN cDNA 1700029I01/similar to RIKEN cDNA 1700029I01 (similar to zinc finger protein 665)	0.9	1424784_at
Klf9	Kruppel-like factor 9	0.9	1428288_at
Prdx2	peroxiredoxin 2	0.9	1430979_a_at
Tcrg	T-cell receptor gamma chain, variable 4	0.9	1422188_s_at
Clec4n	C-type lectin domain family 4, member n	0.9	1425951_a_at
C1qg	complement component 1, q subcomponent, gamma polypeptide	0.9	1449401_at
**Tlr1**	toll-like receptor 1	0.9	1449049_at
**Trim12**	tripartite motif protein 12	0.9	1437432_a_at
**Trim34/LOC434218**	tripartite motif protein 34/similar to tripartite motif protein TRIM34 alpha	0.8	1424857_a_at
Wars	tryptophanyl-tRNA synthetase	0.8	1415694_at
**ifi203**	interferon-activatable gene 203	0.8	1426906_at
**Ndel1**	nuclear distribution gene E-like homolog 1 (A. nidulans)	0.8	1424893_at

Affymetrix GeneChip Mouse Expression Array 430A

a **Bold gene title**: Differential expression verified in an independent analysis, see text

b Mean SLR: Signal log ratio, log_2_

**Table 2:**
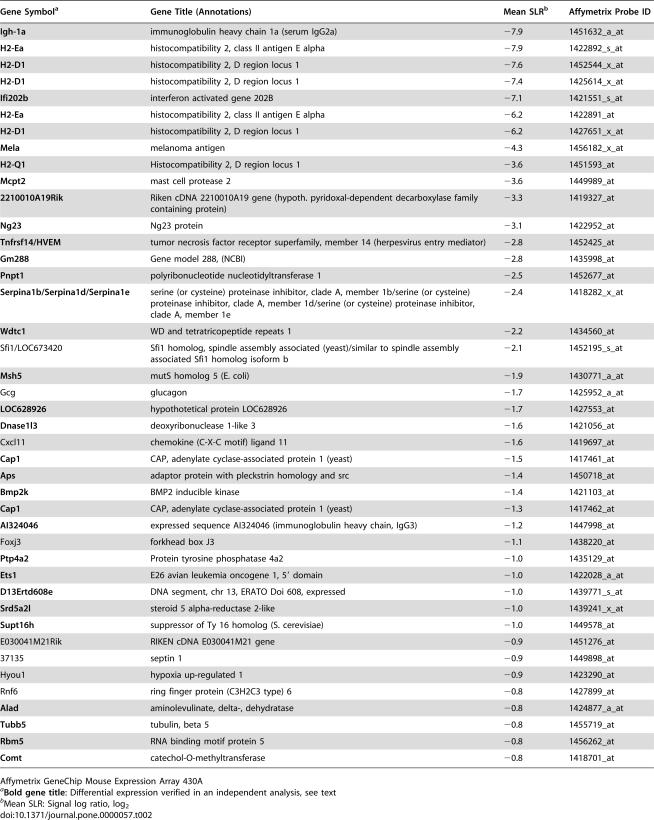
Transcripts with decreased expression in spleens of *T. cruzi*- infected susceptible B6 mice with respect to resistant F1 mice.

Gene Symbol^a^	Gene Title (Annotations)	Mean SLR^b^	Affymetrix Probe ID
**Igh-1a**	immunoglobulin heavy chain 1a (serum IgG2a)	−7.9	1451632_a_at
**H2-Ea**	histocompatibility 2, class II antigen E alpha	−7.9	1422892_s_at
**H2-D1**	histocompatibility 2, D region locus 1	−7.6	1452544_x_at
**H2-D1**	histocompatibility 2, D region locus 1	−7.4	1425614_x_at
**Ifi202b**	interferon activated gene 202B	−7.1	1421551_s_at
**H2-Ea**	histocompatibility 2, class II antigen E alpha	−6.2	1422891_at
**H2-D1**	histocompatibility 2, D region locus 1	−6.2	1427651_x_at
**Mela**	melanoma antigen	−4.3	1456182_x_at
**H2-Q1**	Histocompatibility 2, D region locus 1	−3.6	1451593_at
**Mcpt2**	mast cell protease 2	−3.6	1449989_at
**2210010A19Rik**	Riken cDNA 2210010A19 gene (hypoth. pyridoxal-dependent decarboxylase family containing protein)	−3.3	1419327_at
**Ng23**	Ng23 protein	−3.1	1422952_at
**Tnfrsf14/HVEM**	tumor necrosis factor receptor superfamily, member 14 (herpesvirus entry mediator)	−2.8	1452425_at
**Gm288**	Gene model 288, (NCBI)	−2.8	1435998_at
**Pnpt1**	polyribonucleotide nucleotidyltransferase 1	−2.5	1452677_at
**Serpina1b/Serpina1d/Serpina1e**	serine (or cysteine) proteinase inhibitor, clade A, member 1b/serine (or cysteine) proteinase inhibitor, clade A, member 1d/serine (or cysteine) proteinase inhibitor, clade A, member 1e	−2.4	1418282_x_at
**Wdtc1**	WD and tetratricopeptide repeats 1	−2.2	1434560_at
Sfi1/LOC673420	Sfi1 homolog, spindle assembly associated (yeast)/similar to spindle assembly associated Sfi1 homolog isoform b	−2.1	1452195_s_at
**Msh5**	mutS homolog 5 (E. coli)	−1.9	1430771_a_at
Gcg	glucagon	−1.7	1425952_a_at
**LOC628926**	hypothotetical protein LOC628926	−1.7	1427553_at
**Dnase1l3**	deoxyribonuclease 1-like 3	−1.6	1421056_at
Cxcl11	chemokine (C-X-C motif) ligand 11	−1.6	1419697_at
**Cap1**	CAP, adenylate cyclase-associated protein 1 (yeast)	−1.5	1417461_at
**Aps**	adaptor protein with pleckstrin homology and src	−1.4	1450718_at
**Bmp2k**	BMP2 inducible kinase	−1.4	1421103_at
**Cap1**	CAP, adenylate cyclase-associated protein 1 (yeast)	−1.3	1417462_at
**AI324046**	expressed sequence AI324046 (immunoglobulin heavy chain, IgG3)	−1.2	1447998_at
Foxj3	forkhead box J3	−1.1	1438220_at
**Ptp4a2**	Protein tyrosine phosphatase 4a2	−1.0	1435129_at
**Ets1**	E26 avian leukemia oncogene 1, 5′ domain	−1.0	1422028_a_at
**D13Ertd608e**	DNA segment, chr 13, ERATO Doi 608, expressed	−1.0	1439771_s_at
**Srd5a2l**	steroid 5 alpha-reductase 2-like	−1.0	1439241_x_at
**Supt16h**	suppressor of Ty 16 homolog (S. cerevisiae)	−1.0	1449578_at
E030041M21Rik	RIKEN cDNA E030041M21 gene	−0.9	1451276_at
37135	septin 1	−0.9	1449898_at
Hyou1	hypoxia up-regulated 1	−0.9	1423290_at
Rnf6	ring finger protein (C3H2C3 type) 6	−0.8	1427899_at
**Alad**	aminolevulinate, delta-, dehydratase	−0.8	1424877_a_at
**Tubb5**	tubulin, beta 5	−0.8	1455719_at
**Rbm5**	RNA binding motif protein 5	−0.8	1456262_at
**Comt**	catechol-O-methyltransferase	−0.8	1418701_at

Affymetrix GeneChip Mouse Expression Array 430A

a **Bold gene title**: Differential expression verified in an independent analysis, see text

b Mean SLR: Signal log ratio, log_2_

Over all, only about 0.3% of transcripts present on the microarray were found to be differentially expressed between the two mouse strains. Among these were a number of genes that are involved in immune responses or inflammatory processes, such as: the T cell receptor (TCR) Vβ 13; the TCR gamma chain; osteopontin (*Opn*, or *Spp1*, secreted phosphoprotein 1); complement component 1 subcomponents; the herpes virus entry mediator (*Hvem*), a member of the TNF receptor superfamily and the ligand of the B and T lymphocyte attenuator (BTLA) [32]; *Cxcl11*, a T cell recruiting CXC chemokine (also known as I-TAC); Toll-like receptor 1 (*Tlr1*); *Clec4n*, a C type lectin-like receptor (also termed dectin-2). Among the transcripts with decreased expression, the strongest differences were expectedly found for genes not encoded in the genome of B6 mice, such as *Igh-1a* (an allotype of the *γ2a* gene encoding the heavy chain of Ig2a immunoglobulins, [33]), *H2-Ea* (encoding H2-Eα, an MHC class II protein) [34], [35], and *H2-D1* (encoding an MHC class Ia protein not expressed in mice with the H2^b^ haplotype [36]).

Only a few differentially transcribed genes map to the susceptibility loci on Chromosomes 5 and 17 (table 3). Three transcripts map to the locus on Chromosome 5, with two (the IFN-inducible chemokine *Cxcl11*, and *Bmp2k*, bone morphogenetic protein 2-inducible kinase) being down-regulated, and one (*Spp1*) being upregulated, in susceptible mice. Expression of *Bmp2k* and *Spp1* transcripts were concordantly regulated in the second microarray experiment. *Cxcl11* mRNA was not detectable in either pool of RNA in this experiment, but it was found to be about four-fold decreased in infected B6 mice in an RT-PCR assay (O. Steinmetz, personal communication), corroborating the results of the gene array in which a signal log ratio of −1.6 was determined (corresponding to a three-fold decrease, table 3). By contrast, expression levels of two other CXC chemokines (*Cxcl9* and *Cxcl10*) did not vary between mice in either analysis.

**Table 3:**
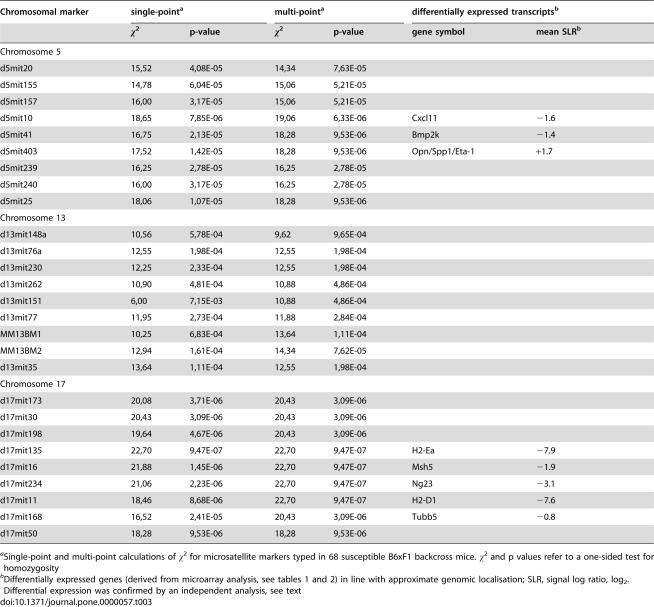
susceptibility loci in B6 mice, and transcripts of genes encoded in these genomic regions that are differentially expressed between *T. cruzi*- infected B6 and F1 mice.

Chromosomal marker	single-point^a^		multi-point^a^		differentially expressed transcripts^b^	
	χ^2^	p-value	χ^2^	p-value	gene symbol	mean SLR^b^
Chromosome 5						
d5mit20	15,52	4,08E-05	14,34	7,63E-05		
d5mit155	14,78	6,04E-05	15,06	5,21E-05		
d5mit157	16,00	3,17E-05	15,06	5,21E-05		
d5mit10	18,65	7,85E-06	19,06	6,33E-06	Cxcl11	−1.6
d5mit41	16,75	2,13E-05	18,28	9,53E-06	Bmp2k	−1.4
d5mit403	17,52	1,42E-05	18,28	9,53E-06	Opn/Spp1/Eta-1	+1.7
d5mit239	16,25	2,78E-05	16,25	2,78E-05		
d5mit240	16,00	3,17E-05	16,25	2,78E-05		
d5mit25	18,06	1,07E-05	18,28	9,53E-06		
Chromosome 13						
d13mit148a	10,56	5,78E-04	9,62	9,65E-04		
d13mit76a	12,55	1,98E-04	12,55	1,98E-04		
d13mit230	12,25	2,33E-04	12,55	1,98E-04		
d13mit262	10,90	4,81E-04	10,88	4,86E-04		
d13mit151	6,00	7,15E-03	10,88	4,86E-04		
d13mit77	11,95	2,73E-04	11,88	2,84E-04		
MM13BM1	10,25	6,83E-04	13,64	1,11E-04		
MM13BM2	12,94	1,61E-04	14,34	7,62E-05		
d13mit35	13,64	1,11E-04	12,55	1,98E-04		
Chromosome 17						
d17mit173	20,08	3,71E-06	20,43	3,09E-06		
d17mit30	20,43	3,09E-06	20,43	3,09E-06		
d17mit198	19,64	4,67E-06	20,43	3,09E-06		
d17mit135	22,70	9,47E-07	22,70	9,47E-07	H2-Ea	−7.9
d17mit16	21,88	1,45E-06	22,70	9,47E-07	Msh5	−1.9
d17mit234	21,06	2,23E-06	22,70	9,47E-07	Ng23	−3.1
d17mit11	18,46	8,68E-06	22,70	9,47E-07	H2-D1	−7.6
d17mit168	16,52	2,41E-05	20,43	3,09E-06	Tubb5	−0.8
d17mit50	18,28	9,53E-06	18,28	9,53E-06		

a Single-point and multi-point calculations of χ^2^ for microsatellite markers typed in 68 susceptible B6xF1 backcross mice. χ^2^ and p values refer to a one-sided test for homozygosity

b Differentially expressed genes (derived from microarray analysis, see tables 1 and 2) in line with approximate genomic localisation; SLR, signal log ratio, log_2_. Differential expression was confirmed by an independent analysis, see text

Five transcripts that were significantly reduced in B6 mice map to the locus on Chromosome 17, which covers the murine MHC complex (table 3). All differences were confirmed in the second microarray experiment with pooled RNA. One additional gene (*Glo1*, glyoxalase 1) close to the locus on Chromosome 17 was insignificantly (SLR−0.6, equivalent to a 1.5 fold decrease) but consistently regulated in that it was decreased in all 9 comparisons between the 3 individual mice of either strain, and the regulation was observed for several transcripts of the gene. It maps centromerically to the suceptibility locus at about 16 cM.

No significant expression differences were found to map to the putative locus on Chromosome 13 (cytogenetic band 13D2.3), but three transcripts of an expressed gene (4833420G17Rik) were reproducibly regulated below the threshold (SLR+0.4, eqivalent to a 1.3 fold increase). It encodes a protein with a gonadotropin beta chain-like domain. The slightly increased expression in B6 mice was confirmed in the pooled RNA microarray analysis. Another transcript from this locus, *Paip1* (polyadelynate binding protein-interacting protein 1, a translation factor), was downregulated in 5 of 9 comparisons only (SLR−1.9), but this could not be substantiated in the second experiment.

Numerous gene transcripts have been shown to be involved in immunity of Chagas' disease and its experimental models. A few of these displayed differential expression below the threshold level, and there was concordant regulation in the microarray experiment with pooled RNA samples. Several transcripts of inducible TGF-β were increased in B6 mice (SLR+0.6). This is noteworthy as it supports results of a previous investigation on the differences of the immune response of B6 and F1 mice to *T. cruzi* in which increased TGF-β expression was shown to enhance susceptibilty [28]. Transcription signals of *CD44* (coding for an adhesion factor on activated T and B cells; SLR+0.4), and of *granzyme B* (SLR+0.4) were slightly upregulated in B6 mice. A downregulated transcription signal in B6 mice was found for the beta chain of the IL-2 receptor (SLR−0.5).

## Discussion

Disease following infection with a pathogen is the result of a complex interaction between the host and the infectious agent [37]. Both host and pathogen genomes are involved in determining the severity of a sickness, and enviromental factors also contribute to the course of an ailment. Genetic variation within human cohorts complicates matters with respect to the identification of genetic sources for disease severity, and in general, several gene loci, splice variants and variation of gene copy numbers are involved [38]. It is thus conceivable that the identification of suceptibility genes to infectious diseases in man has not been fruitful thus far, despite of the progress that genome sequencing has achieved. Therefore, it may be a more straightforward approach to pinpoint genes that affect the severity of infectious disease in animal models, and then to assess whether orthologous mechanisms are at work in the human situation [39]. Since a hosts genotype manifests via transcription of genes, candidate genes may be identified by a combination of mapping data and transcriptional profiling. Determining the gene expression differences from susceptibility loci is thus a hypothesis-driven experiment with the advantage that the multiple testing problem of microarray analyses disappears and that the verification of the gene expression data by independent methods is not strictly required [40].

Morphological and cytometrical analysis of the organ with the greatest differences in parasite loads, the spleen, revealed a disruption of normal anatomical structure and modulation of cellular composition in susceptible B6 mice. In addition, a substantial increase of apoptotic cells was observed, possibly accounting further for the increased parasite burden, since the presence of apoptotic cells were connected with increased parasite proliferation, and with decreased trypanocidal activity of infected macrophages [29]. B cell apoptosis has also been shown to correlate with reduced numbers of B cells and with a suppressed immune response to *T. cruzi* [41]. The loss of B cells and of splenic follicles make a decreased humoral immune response to the parasite a candidate pathogenetic pathway. Indeed, on day 17 of infection, serologic reactivity to parasite antigens was decreased in B6 mice for antibodies of all IgG subclasses, except for IgG2b, when compared with F1 mice (unpublished observation). The requirement of an effective humoral response for the resolution of experimental *T. cruzi* infection has been demonstrated [42]–[44]. The bias towards macrophages at the expense of B cells in the spleen of susceptible mice reproduces results of a previous study with parasites of differential virulence. In this study, virulent parasites, but not avirulent ones, caused a reduction of CD19+ B cells, and an increase of Mac3+ macrophages, in the spleen of infected mice [45].

We analysed genome wide expression in the spleen, prior to an insufficient immune response to the parasite, and prior to the profound alterations in cellular composition, in order to identify transcriptional correlates of susceptibility that mapped to the genomic susceptibility loci. We identified three candidate genes from the susceptibility locus on Chromosome 5, and five from the locus on Chromosome 17, with significantly different expression between differentially resistant strains of mice. The organisation of secondary lymphoid organs, such as the spleen, is regulated by compartmentalised expression of lymphoid tissue chemokines, enabling effective communication between lymphocytes and antigen-presenting cells to mount protective immune responses to pathogens. It was therefore interesting to find that the expression of T cell attractant CXC chemokine ligand *Cxcl11* mRNA was decreased in suscptible mice. CXCL11 is the most potent agonist of the chemokine receptor CXCR3 that is predominantly found on activated T cells, Th1 type T cells, peripheral T cells and a subset of B cells [46], [47]. It is produced by monocytoid cells, epithelial cells, neutrophils and endothelial cells, and its expression is induced by IFN-γ. It is involved in generating Th1 type immune responses. CXCL11 was also reported to be a natural antagonist of CCR3 [48], [49] and of CCR5 [47], indicating a possible role in counteracting the effects of other inflammatory chemokines such as RANTES, MIP-1α and MIP-1β. More recently, CCR5 expression was shown to augment the inflammatory reaction and to participate in host protection in experimental Chagas' disease [50], [51]. It was now published that CXCL11 also binds to a newly identified chemokine receptor, CXCR7, that is expressed on activated endothelial and other cells, providing a growth and survival advantage and adhesion properties [52]. The role of CXCL11 in *T. cruzi* infection has not been investigated, but the two other CXCR3- binding chemokines, CXCL9 and CXCL10, were found to contribute to a protective immune response in mice [53], [54]. Decreased expression of *Cxcl11* mRNA in susceptible mice may be involved in the insufficient immune response observed in B6 mice by preventing adequate association between T cells and antigen-presenting cells and/or B cells, or by reduced antagonism of CCR3- and/or CCR5- mediated inflammatory reactions.

A further gene from the locus on Chromosome 5 with decreased expression in B6 mice was *Bmp2k*. Bone morphogenetic proteins play a role in skeletal development. Signalling involves transcription of *Bmp2k* which encodes a serin/threonin kinase. Activation of BMP2K attenuates osteocalcin expression and reduces osteoblast differentiation [55]. Its role in infections in general, and its possible role in determing the outcome of experimental *T. cruzi* infection, remain to be determined.

Osteopontin (OPN; also termed secreted phosphoprotein 1, SPP1, or early T lymphocyte activation 1, ETA-1) was primarily described as an early adhesion receptor expressed after T cell activation and mediating macrophage migration [56], [57]. It is regarded as an essential factor in inducing Th1 type immune responses and has been described as a regulator of resistance to microbial infection [58]–[61]. Recently, its role in Th1 type viral immunity was disputed [62], and it was shown that SPP1 mediated IFN-α production in plasmacytoid dendritic cells [63]. Several cells express *Spp1* following viral or bacterial infection, including dendritic cells, T cells and macrophages, and it functions as a cytokine and anti-apoptotic growth factor, involved in adhesion, chemotaxis, cell differentiation and inflammation. Osteopontin was also found to participate in collagen fibrillogenesis and matrix re-organisation in wound healing [64]. Transcription of *Spp1* was increased in *T. cruzi* susceptible B6 mice, indicating its role in determining the strong Th1 type immune response. Possibly, its expression is increased secondary to the severe disruption of splenic architecture and due to an increased re-modelling activity.

Several genes within the susceptibility locus on Chromosome 17 were expressed at reduced levels in susceptible mice. The MHC H2-D1 and H2-Eα genes are not encoded in B6 mice and signals were thus drastically decreased. MHC encoded proteins are essential for the presentation of antigens to T lymphocytes. MHC polymorphism is believed to be driven by pathogen-based mechanisms [65], and it has been associated with increased resistance to a model infection [66]. The heterozygosity of the intercross F1 at the MHC locus (H2^b/d^) implies that a greater number of antigens can potentially be presented to antigen-specific T cells, resulting in a larger pool of lymphoytes that are able to respond to the pathogen. Since a protective response to *T. cruzi* infection requires both cellular and humoral acquired immune effector mechanisms, it follows that both MHC class I and class II genes are strong candidates for determining susceptibility. However, the presence of a heterozygous state as such did not necessarily confer resistance [14]. Certain H2 haplotypes were associated with a protective effect under given experimental conditions (reviewed by Campbell [3]). In the present study, though, both parental strains succumbed to infection [15], [30], and neither H2 haplotype (neither H2^b^ nor H2^d^) was thus protective in itself. In man, the influence of HLA haplotypes on disease progression has been demonstrated [6], [67], [68].

The other genes with decreased expression within the locus on Chromosome 17 have not been assigned a role in immunity so far. *Tubb5* codes for the β5 isotype of the β-tubulin family and is expressed at low levels ubiquitously [69]. *Msh5* codes for a member of the mismatch repair family of proteins, that is expressed mainly in gonads and has a role in chromosome pairing during meiosis; deficiency was associated with apoptosis of testicular and ovarian cells and sterility [70], [71]. The expressed sequence *Ng23* has not yet been classified regarding function or process [72].

The immunogenetic background of susceptibility of inbred mice to experimental infections in general, and to experimental infection with *T. cruzi* in particular, has not been delineated despite progress in analysing numerous immunologic players and pathways. The present work aimed at identifying candidates that direct the many secondary variations of the immune response that have been described. Further work is needed to substantiate these proposals and to clarify the role of susceptibility genes in the evolution of an ineffective immune response. It is important to recognise that the results are strictly related to the common model that has been used in this study. It is certainly probable that alternate mechanisms apply in other inbred mice and with other stocks and strains of *T. cruzi*.

## Materials and Methods

### Parasites and mice

The *T. cruzi* tulahuen strain was used in all experiments. Mouse experiments were registered at and approved by the Federal Health Authorities of the State of Hamburg. Parasites were maintained in Balb/c mice by fortnightly passage. Trypomastigotes were counted in a Neubauer chamber following lysis in a solution of NH_4_Cl (0.89% w/v) [73], and blood was appropriately diluted with phosphate buffered saline (PBS) for an experimental infection with 10^4^ parasites. C57BL/6 (B6) and C57BL/6xDBA/2 (B6D2F1, F1) mice were obtained from Charles River (Sulzbach, Germany) and infected at the age of 6–8 weeks by inoculation of parasites into the peritoneum in a volume of 200 µl. Parasitaemia was determined in 2 µl of tail vein blood after lysis in 18 µl of NH_4_Cl.

### Quantitative PCR

Relative tissue parasite burdens were determined by real time PCR in specimens of about 20 mg of tissue. DNA extraction was performed with a Gentra Puregene™ tissue kit (Biozym, Hessisch Oldendorf, Germany) according to the manufacturer's instructions. DNA was dissolved in 50 µl of H_2_O, and a 100 fold dilution was used for amplification. *T. cruzi* PCR was performed on an Abi Prism 7700 SDS Instrument (Applied Biosystems, Weiterstadt, Germany) as previously described [30]. The quantity of host DNA was determined by real time PCR of β-actin from the same samples as described [30], with the exception that thermal cycles were 20 s at 95°C and 40 s at 58°C. The quantity of parasite DNA in a given sample is expressed in relation to its content of β-actin DNA.

### Histology

Specimens from the spleen were obtained from naive and from experimentally infected mice, fixed in buffered 10% formalin and embedded in paraffin. Sections were stained with hematoxylin-eosin (HE) and examined by light microscopy.

### Immunohistochemistry

Spleen specimens were shock frozen in liquid nitrogen and stored at −80°C until further processing. Sections of 5–8 µm were stained with one of the following antibodies (obtained from Beckton Dickinson, Heidelberg, Germany): anti-CD4; anti-CD8; or anti-CD19; antibodies were diluted 1∶50 in PBS. Sections were then fixed in paraformaldehyde, and a secondary biotinylated anti-rat Ig antibody was applied for 30 min. Detection was achieved with streptavidin/horseradish peroxidase conjugate and developed with the peroxidase substrate diaminobenzidine (Dako, Hamburg, Germany). The TUNEL reaction on some sections was performed with the ‘In Situ Cell Death Detection Kit, AP’ according to the manufacturers instructions (Roche, Mannheim Germany) Sections were counterstained with hematoxylin and analysed by optical microscopy.

### Flow cytometry

Spleen cell surface expression was analysed by staining of cell suspensions with antibodies against either CD4 (Cy5-conjugated) and CD8 (FITC-conjugated), against CD19 (PE-conjugated), or against CD11b (PE-conjugated). Some samples were stained with Annexin V and with propidium iodide to determine fractions of apoptotic cells. All reagents were obtained from Becton Dickinson (Heidelberg, Germany). Cells were fixed in 1% paraformaldehyde and analysed with a FACSCalibur flow cytometer (Beckton Dickinson, Mountain View, CA, USA) using the CellQuest software. Results are expressed as the percentage of cells analysed that were positive for the surface marker of interest.

### Microsatellite genotyping

As an extension of our previous study [30], a further 22 male backcross mice (B6xF1) were experimentally infected and identified as being susceptible to a lethal outcome. Genomic DNA of these animals was subjected to microsatellite genotyping to confirm and fine map the genomic regions on Chromosomes 17, 5, and 13, previously identified as putatively linked to susceptibility. Nine polymorphic markers for each region were analysed, as described elsewhere [30]. The markers MM13BM1 and MM13BM2 were identified on mouse Chromosome 13 and amplified with the follwing primer pairs: MM13BM1F (5′atagccaccaacaggcattg) and MM13BM1R (5′FAM-tcggctttccctaaactgtg) as well as MM13BM2F (5′gcttaggcaatgggtctgatag) and MM13BM2R (5′HEX-tttgggtttgtatatgtctgactg). For statistical analysis, genotyping data of the newly typed 22 animals were combined with the original data set obtained from 46 susceptible mice in the previous study. Chi-square statistics were computed comparing the numbers of homozygous versus heterozygous mice. The construction of the genetic maps was performed with MAPMAKER/EXP v3.0b.

### Microarray Analysis

Procedures for cDNA synthesis, labelling and hybridization were carried out according to the manufacturer's protocol (Affymetrix). All experiments were performed using Affymetrix mouse genome GeneChip 430A. Three mice of either strain (B6 and F1, respectively) from three independent experiments were sacrificed on day 11, the spleen was removed, and single cell suspensions were prepared. All procedures were performed at 4°C. RNA was prepared with a Qiagen RNeasy kit (Qiagen, Hilden, Germany) according to the manufacturers instructions, and the quality of the preparation was checked by agarose gel electrophoresis. In brief, 5 µg of total RNA were used for first strand cDNA synthesis with an HPLC-purified T_7_-(dT)_24_ primer (One-Cycle cDNA Synthesis Kit, Affymetrix). Synthesis of biotin-labeled cRNA was carried out using the IVT Labeling Kit (Affymetrix) and then purified (Sample Cleanup Module, Affymetrix). For hybridization, 15 µg of fragmented cRNA was incubated with the chip in 200 µl of hybridization solution in Hybridization Oven 640 (Affymetrix) at 45°C for 16 hours. GeneChips were then washed and stained using the Affymetrix Fluidics Station according to the GeneChip Expression Analysis Technical Manual. Microarrays were scanned with the Agilent GeneArray Scanner, and the signals were processed using the GeneChip expression analysis algorithm (v.2; Affymetrix). To compare samples and experiments, the trimmed mean signal of each array was scaled to a target intensity of 100. Absolute and comparison analyses were performed with Affymetrix MAS (version 5.0, Affymetrix) software using default parameters. To assist in the identification of genes that were positively or negatively regulated in the experiment, we selected genes that showed a signal log ratio of at least 0.8 (increase) or −0.8 (decrease) compared to the baseline (B6 compared to F1) [74]. Annotations were further analyzed with interactive query analysis at www.affymetrix.com. Pathways and other functional groupings of genes were evaluated for differential regulation using the visualisation tool GenMAPP (UCSF) as described elsewhere [75], [76].
